# Chl1 coordinates with H3K9 methyltransferase Clr4 to reduce the accumulation of RNA-DNA hybrids and maintain genome stability

**DOI:** 10.1016/j.isci.2022.104313

**Published:** 2022-04-27

**Authors:** Deyun He, Yazhen Guo, Jinkui Cheng, Yu Wang

**Affiliations:** 1 College of Biological Sciences, China Agricultural University, Beijing, 100193, China; 2 College of Bioengineering, Key Laboratory of Shandong Microbial Engineering, State Key Laboratory of Biobased Material and Green Papermaking, Qilu University of Technology, Shandong Academy of Sciences, Jinan, Shandong 250353, China

**Keywords:** Molecular biology, Cell biology

## Abstract

A genome-wide analysis in *Schizosaccharomyces pombe* indicated that double-deletion mutants of Chl1 and histone H3K9 methyltransferase complex factors are synthetically sick. Here, we show that loss of Chl1 increases the accumulation of RNA-DNA hybrids at pericentromeric dg and dh repeats in the absence of the H3K9 methyltransferase Clr4, which leads to genome instability, including more severe defects in chromosome segregation and increased chromatin accessibility. Localization of Chl1 at pericentromeric regions depends on a subunit of replication protein A (RPA), Ssb1. In wild-type (WT) cells, transcriptionally repressed heterochromatin prevents the formation of RNA-DNA hybrids. When Clr4 is deleted, dg and dh repeats are highly transcribed. Then Ssb1 associates with the displaced single-stranded DNA (ssDNA) and recruits Chl1 to resolve the RNA-DNA hybrids. Together, our data suggest that Chl1 coordinates with Clr4 to eliminate RNA-DNA hybrids, which contributes to the maintenance of genome integrity.

## Highlights

• Double mutant of Chl1 and Chl1 leads to the accumulation of RNA-DNA hybrids
• RNA-DNA hybrids at pericentromeric regions affect genome stability and cell viability
• Ssb1 recruits Chl1 to unwind RNA-DNA hybrids in the absence of Clr4

## Graphical abstract

**Figure:**
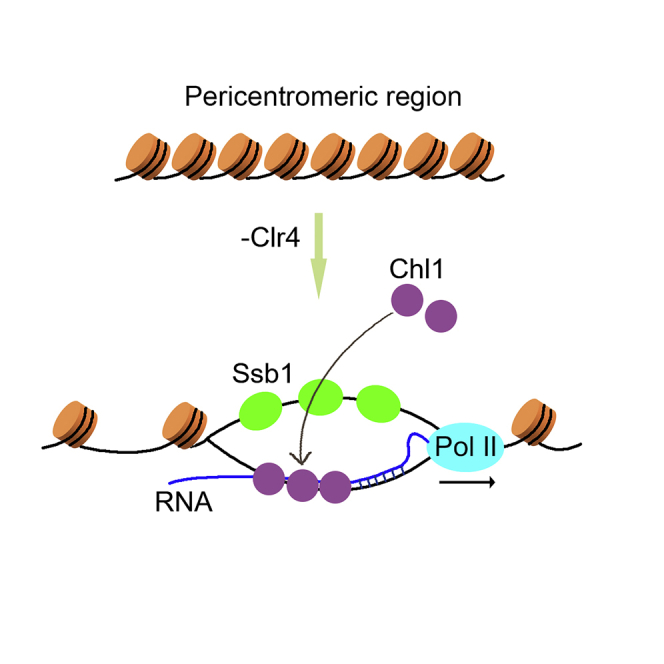

## Introduction

Fission yeast pericentromeric heterochromatin contains highly repetitive *dg* and *dh* repeats that are characterized by condensed nucleosome arrays, transcriptionally inert regions, and histone H3K9 methylation (Grewal and Jia, 2007). This pericentromeric heterochromatin mediates sister chromatid cohesion by recruiting cohesin, a multiprotein complex, through Swi6 that recognizes methylated H3K9 specifically (Bernard et al., 2001). The association of sister chromatids ensures their segregation at anaphase. Accordingly, disruption of heterochromatin by deleting its regulators, such as histone H3K9 methyltransferase Clr4 and RNAi components, leads to chromosome segregation defects (Ekwall et al., 1996; Ellermeier et al., 2010). Moreover, depletion of H3K9 methylation by mutating Clr4 results in the release of transcriptional silence at heterochromatic regions (Ekwall et al., 1996).

In wild-type cells of fission yeast, noncoding RNAs transcribed from *dg* and *dh* repeats at heterochromatic regions can generate RNA-DNA hybrids (Nakama et al., 2012), but these regions are specifically transcribed at S phase and then repressed by the reestablishment of heterochromatin (Chen et al., 2008). A genome-wide mapping of RNA-DNA hybrids indicates that they are distributed at rDNAs, transposons, a subset of open reading frames (ORFs), and tRNAs (Chan et al., 2014; El Hage et al., 2014). Although RNA-DNA hybrid plays a positive role in DNA repair (Keskin et al., 2014; Ohle et al., 2016), it is also a threat to genome stability (Hamperl and Cimprich, 2014). One of the possible reasons is that the cotranscriptional RNA-DNA hybrid can form a three-stranded R-loop structure with the displaced single-stranded DNA (ssDNA). R-loops have physiological functions in transcriptional regulation, recombination, and DNA repair (Garcia-Muse and Aguilera, 2019; Niehrs and Luke, 2020; Santos-Pereira and Aguilera, 2015; Sun et al., 2013). Nonetheless, there is also considerable evidence showing that misregulation of R-loop causes hyperrecombination, transcription-replication conflicts, and DNA damage (Costantino and Koshland, 2018; Crossley et al., 2019; Hamperl et al., 2017; Skourti-Stathaki and Proudfoot, 2014), which may lead to neurological diseases, cancer, or other clinical disorders in humans. A range of factors have been found to process or resolve RNA-DNA hybrids, which protect against genome instability caused by the accumulation of RNA-DNA hybrids. On the basis of their biochemical activities, RNase H enzymes and helicases are two types of factors known to remove RNA-DNA hybrids. RNase H enzymes hydrolyze the RNA in RNA-DNA hybrid (Cerritelli and Crouch, 2009; Nowotny et al., 2005; Rychlik et al., 2010), whereas RNA/DNA helicases can unwind this structure (Kim et al., 1999; Song et al., 2017). Studies have shown that the *rnh1Δ rnh201Δ* double mutant of fission yeast, deficient in two RNase H enzymes, exhibits disruption in DNA replication (Zhao et al., 2018). Mutation of the budding yeast helicase Sen1 causes defects in transcript termination and hyperrecombination due to RNA-DNA hybrid accumulation (Mischo et al., 2011). The yeast RECQ-like helicase Sgs1, as well as its human homolog BLM, can repress R-loop accumulation and maintain genome stability owing to its function in resolution of RNA-DNA hybrids (Chang et al., 2017).

An ever-growing number of helicases that take part in RNA-DNA hybrid processing have been identified. However, the molecular mechanisms of some helicases have not been validated, although they exhibit biochemical activity toward RNA-DNA hybrid. For example, biochemical studies have shown that human Chl1R can progress along ssDNA in the 5′-3′ direction and unwind DNA-DNA and RNA-DNA substrates (Hirota and Lahti, 2000). Although the functions of Chl1 in cohesion, DNA repair, and heterochromatin regulation appear to be evolutionarily conserved from budding yeast to mammals (Das and Sinha, 2005; Inoue et al., 2011; Inoue et al., 2007; Laha et al., 2006; Holloway, 2000; Samora et al., 2016; Skibbens, 2004), the link between its biological functions and biochemical activity on RNA-DNA hybrid processing still needs to be investigated. Genome-wide genetic interaction analyses have shown that double mutants of Chl1 and the components of the histone H3K9 methyltransferase complex CLRC, such as Clr4, Raf1, and Rik1, display a synthetically sick phenotype (Roguev et al., 2008; Ryan et al., 2012), indicating that Chl1 might be required for normal growth in the absence of H3K9 methylation, a hallmarker of heterochromatin. It would be interesting to decipher the relationship between Chl1 and members of CLRC complex.

In this study, we found that the fission yeast Chl1 prevents the accumulation of RNA-DNA hybrids generated from heterochromatic regions in the absence of Clr4. Disruption of heterochromatin in Clr4 mutant releases transcriptional silencing, which may increase the formation of RNA-DNA hybrids. Afterward, Ssb1, a subunit of replication protein A (RPA), binds to the displaced ssDNA, and then recruits Chl1 to these regions to resolve RNA-DNA hybrids, which protects genome integrity.

## Results

### *chl1Δ* is synthetically sick with *clr4Δ*

**Figure 1:**
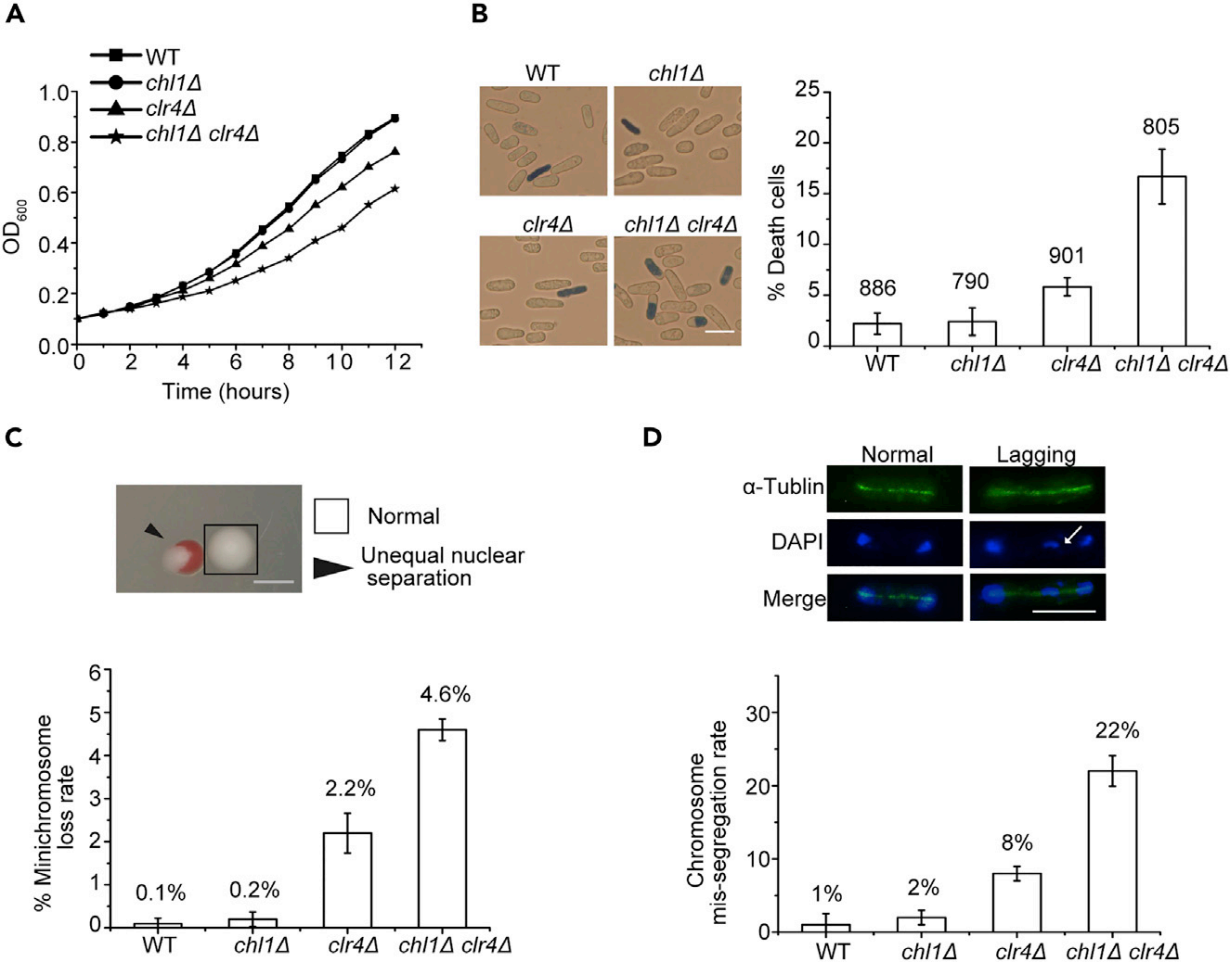
Double deletion of Chl1 and Clr4 decreases cell viability (A) Growth curves of WT, *chl1Δ*, *clr4Δ*, and *chl1Δ clr4Δ* cells. (B) Methylene blue staining of indicated strains (left) and the percentages of dead cells (right). The numbers of dead cells were counted after methylene blue staining for 10 min, and the numbers of total cells were labeled on the top of each column. Scale bar: 10 μm. (C) The loss rate of minichromosome Ch16. Colonies that lost Ch16 at first mitotic division are half red and half white (indicated by arrow). The percentages of minichromosome loss are indicated on the top of each column. Scale bar: 1 mm. (D) Cells were stained with an α-tubulin antibody to visualize tubulin (green) and with DAPI to visualize DNA (blue). Scale bar: 5 μm. The percentages of cells with lagging chromosomes at anaphase are labeled on the top of each column. Lagging chromosome is indicated by arrow. One hundred anaphase cells per strain were counted. Values are displayed as mean ± SEM.

To confirm the negative genetic interaction between Chl1 and Clr4, we constructed single- or double-deletion mutants of Chl1 and Clr4 and compared the growth rate of wild type (WT), *chl1Δ*, *clr4Δ*, and *chl1Δ clr4Δ* cells. The results indicated that the growth curves of WT and *chl1Δ* were comparable, whereas the *clr4Δ* mutant grew slightly slower than WT. Moreover, as expected, a significant growth delay of *chl1Δ clr4Δ* cells was observed relative to WT and the single mutants (Figure 1A). To determine the reason for the slow growth of double mutant, we stained the yeast cells with methylene blue and counted the number of blue cells. Methylene blue can be applied to identify the viability of cells, with live cells reducing the dye, so that only dead cells stain blue. Consistent with the growth curves, the percentage of dead cells of *clr4Δ* was slightly higher than those of WT and *chl1Δ*, whereas blue-stained *chl1Δ clr4Δ* cells were much more abundant than WT cells and the two single mutants (Figure 1B). These data suggest that the growth defect of *chl1Δ clr4Δ* cells might be due to the high percentage of dead cells that cannot proliferate any further.

Heterochromatin is required for cohesion of sister chromatids. Disruption of heterochromatin by deleting genes that affect H3K9 methylation causes improper chromosome segregation during mitosis. Daughter cells without intact chromosomes lose viability. To elucidate whether the low viability of double mutants was due to chromosome missegregation, we grew WT, *chl1Δ*, *clr4Δ*, and *chl1Δ clr4Δ* cells harboring the nonessential minichromosome Ch16 on low-adenine medium and calculated the chromosome loss rates of the four strains (Niwa et al., 1986). The minichromosome Ch16 in WT and *chl1Δ* cells was faithfully segregated, whereas the loss rates increased to 2.2% and 4.6% per division in the *clr4Δ* and *chl1Δ clr4Δ* backgrounds, respectively (Figure 1C). In addition to a high rate of chromosome loss, an elevated incidence of lagging chromosomes is also indicative of defects in chromosome segregation. Spindle microtubules and DNA were stained with an α-tubulin antibody and DAPI, respectively, and visualized by fluorescence microscopy (Figure 1D). We counted 100 cells of each strain during anaphase and found that 1% and 2% of cells displayed lagging chromosomes in WT and the *chl1Δ* mutant, respectively, whereas 8% of *clr4Δ* cells and 22% of *chl1Δ clr4Δ* cells had lagging chromosomes (Figure 1D). These findings suggest that deletion of Chl1 aggravated chromosome segregation defects in the *clr4Δ* mutant, which may have led to the low viability of the double-deletion mutant. Based on the enzymatic activity of ChlR, a human homolog of Chl1, disruption of heterochromatin might bring about a duplex structure that can be resolved by Chl1. Considering that heterochromatin is required for cohesin attachment, we propose that this chromatin structure and dissociation of cohesion synergistically contributed to chromosome segregation defects, leading to a large number of dead cells in *chl1Δ clr4Δ*.

### Chl1 is involved in the repression of RNA-DNA duplexes generated from highly transcribed pericentromeric repeats

**Figure 2:**
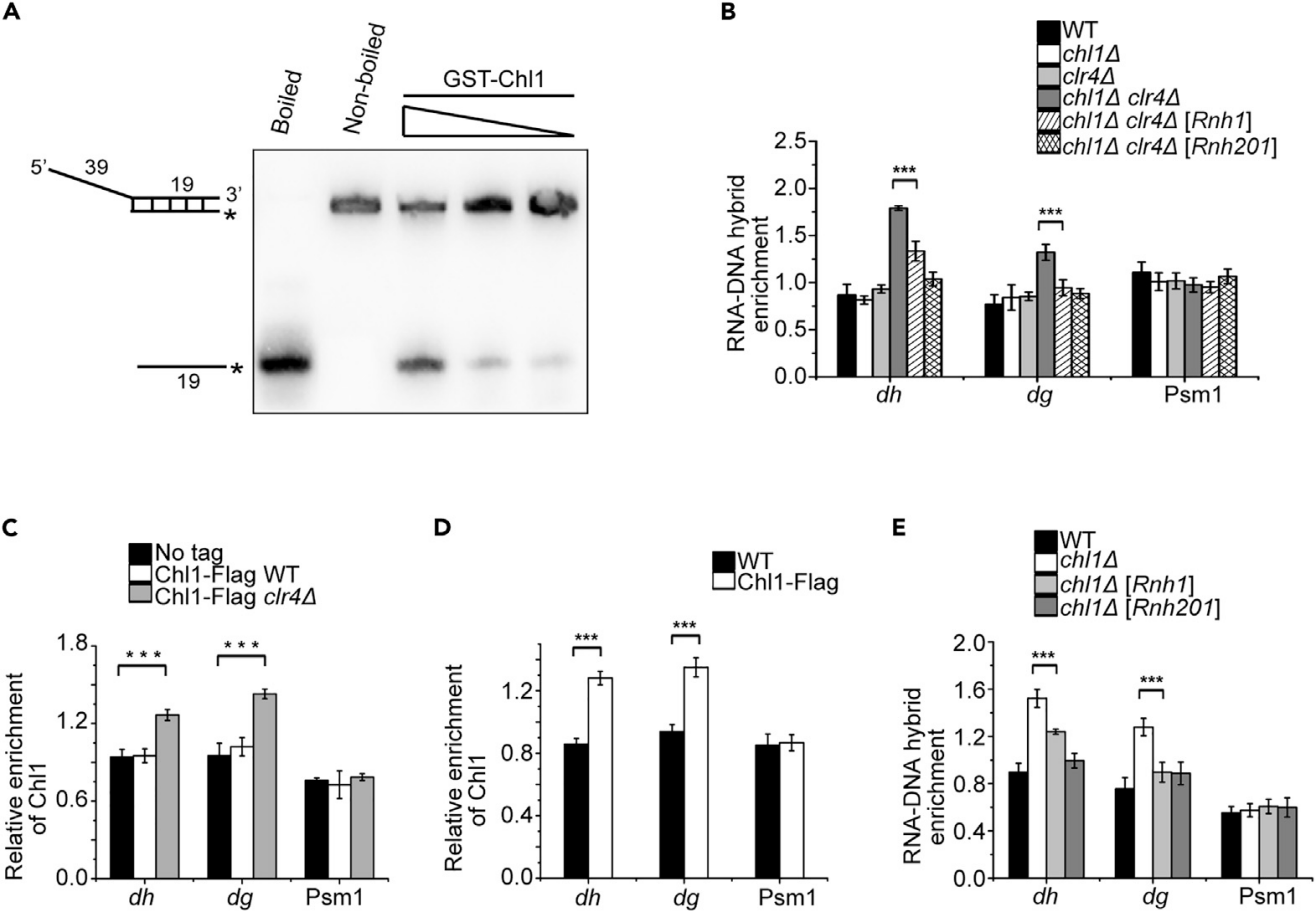
Chl1 represses RNA-DNA hybrids generated from the highly transcribed pericentromeric regions (A) Chl1 has helicase activity on RNA/DNA duplexes. 100 ng, 50 ng, and 25 ng of GST-Chl1 proteins purified from *E. coli* were incubated with 0.05 nM duplex RNA/DNA substrate at 30°C for 10 min, respectively. (B) DRIP-qPCR shows the enrichment of DNA-RNA hybrids in the indicated strains transformed with an empty vector or a vector harboring Rnh1 or Rnh201 at the indicated loci. Error bars represent ±SE of three technical replicates from one DRIP with an S9.6 antibody. Three biological replicates were done with the similar results. (C) ChIP-qPCR shows that Chl1 is enriched at *dg* and *dh* repeats in *clr4Δ* cells. (D) ChIP-qPCR indicates that Chl1 is enriched at *dg* and *dh* repeats in S phase. (E) DRIP-qPCR shows the accumulation of DNA-RNA hybrids in the indicated strains transformed with an empty vector or a vector harboring Rnh1 or Rnh201 at the indicated loci in S phase. Cells were synchronized in S phase with hydroxyurea (HU). Error bars represent ±SE of three technical replicates. The results have been replicated three times. ∗∗∗p < 0.001, Student’s t test.

Previous study showed that human ChlR has helicase activity on RNA-DNA duplexes (Hirota and Lahti, 2000), and we therefore tested whether fission yeast Chl1 can also unwind RNA-DNA hybrids *in vitro*. When an RNA-DNA heteroduplex containing a biotin-labeled RNA oligonucleotide was used as substrate, Chl1 displaced the RNA from the hybrid, indicating that Chl1 of fission yeast has helicase activity on RNA-DNA hybrid (Figure 2A). Depletion of Clr4 releases transcriptional silencing of pericentromeric *dg* and *dh* repeats (Volpe et al., 2002), implying that *clr4Δ* increases the chance of generating cotranscriptional RNA-DNA hybrids at these regions compared with WT cells. To detect the level of RNA-DNA hybrids at pericentromeric regions, we performed DNA-RNA immunoprecipitation (DRIP) using an S9.6 antibody specific for DNA-RNA hybrids. To our surprise, we did not observe the accumulation of RNA-DNA hybrids in WT and the two single mutants, whereas the levels of RNA-DNA hybrid in *chl1Δ clr4Δ* cells at *dg* and *dh* repeats were elevated. At the same time, there were no differences in Psm1 among these strains; Psm1 gene is transcribed at a low level in euchromatic region (Figure 2B). The transcription of *dg* and *dh* repeats in *chl1Δ clr4Δ* slightly decreased as compared with that in *clr4Δ* (Figure S1), suggesting that the enrichment of cotranscriptional RNA-DNA hybrids was not because of the increased transcription in *chl1Δ clr4Δ*, but due to the function of Chl1 in unwinding DNA-RNA hybrids generated from pericentromeric repeats in the absence of Clr4. RNA/DNA helicase and RNase H process RNA-DNA hybrids in different manners. To confirm the accumulation of RNA-DNA hybrids, we overexpressed Rnh1 or Rnh201, two RNase H enzymes of *Schizosaccharomyces pombe*, under the control of an inducible nmt1 promoter in *chl1Δ clr4Δ* cells, and examined the levels of RNA-DNA hybrid at *dg* and *dh* repeats. The DRIP experiment showed that overexpression of Rnh1 or Rnh201 can reduce RNA-DNA hybrids at these regions (Figure 2B). These results suggest that Clr4 and Chl1 can suppress RNA-DNA hybrid formation by repressing transcription and resolving these duplexes, respectively.

The fact that Chl1 can eliminate the formation of RNA-DNA hybrid at pericentromeric regions in the absence of Clr4 prompted us to detect the localization of Chl1 at *dg* and *dh* repeats. We generated strains expressing Chl1-Flag in the WT and *clr4Δ* backgrounds and performed chromatin immunoprecipitation (ChIP) analysis with an antibody against the Flag tag. The ChIP signals of Chl1 were elevated in the absence of Clr4 within *dg* and *dh* repeats, suggesting that Chl1 can be recruited to these regions when transcription was released (Figure 2C).

Next, we wanted to determine whether Chl1 can repress the accumulation of RNA-DNA hybrid in WT background. Pericentromeric repeats are specifically transcribed during S phase in fission yeast (Chen et al., 2008). If Chl1 resolves RNA-DNA hybrids generated from highly transcribed *dg* and *dh* repeats, an increase in the recruitment of Chl1 at these regions should be observed at S phase. To test this hypothesis, we treated WT cells and cells harboring the Flag-tagged Chl1 with hydroxyurea (HU), which can block the cell cycle in S phase by inhibiting ribonucleotide reductase, and then performed ChIP analysis with an anti-Flag antibody. As we expected, Chl1 signal was enhanced at *dg* and *dh* repeats (Figure 2D). Consistent with this, deletion of Chl1 resulted in the accumulation of RNA-DNA hybrids at pericentromeric regions in cells at S phase, whereas overexpression of Rnh1 or Rnh201 reduced their levels (Figure 2E). Taken together, these data indicate that Chl1 plays a role in the suppression of RNA-DNA hybrids produced at pericentromeric regions. Nonetheless, as S phase is a short period during the entire cell cycle and the subsequent reestablishment of heterochromatin suppresses transcription, RNA-DNA hybrids cannot be detected at *dg* and *dh* repeats in asynchronous *chl1Δ* cells.

### Chl1 is required for genome stability in *clr4Δ* cells

**Figure 3:**
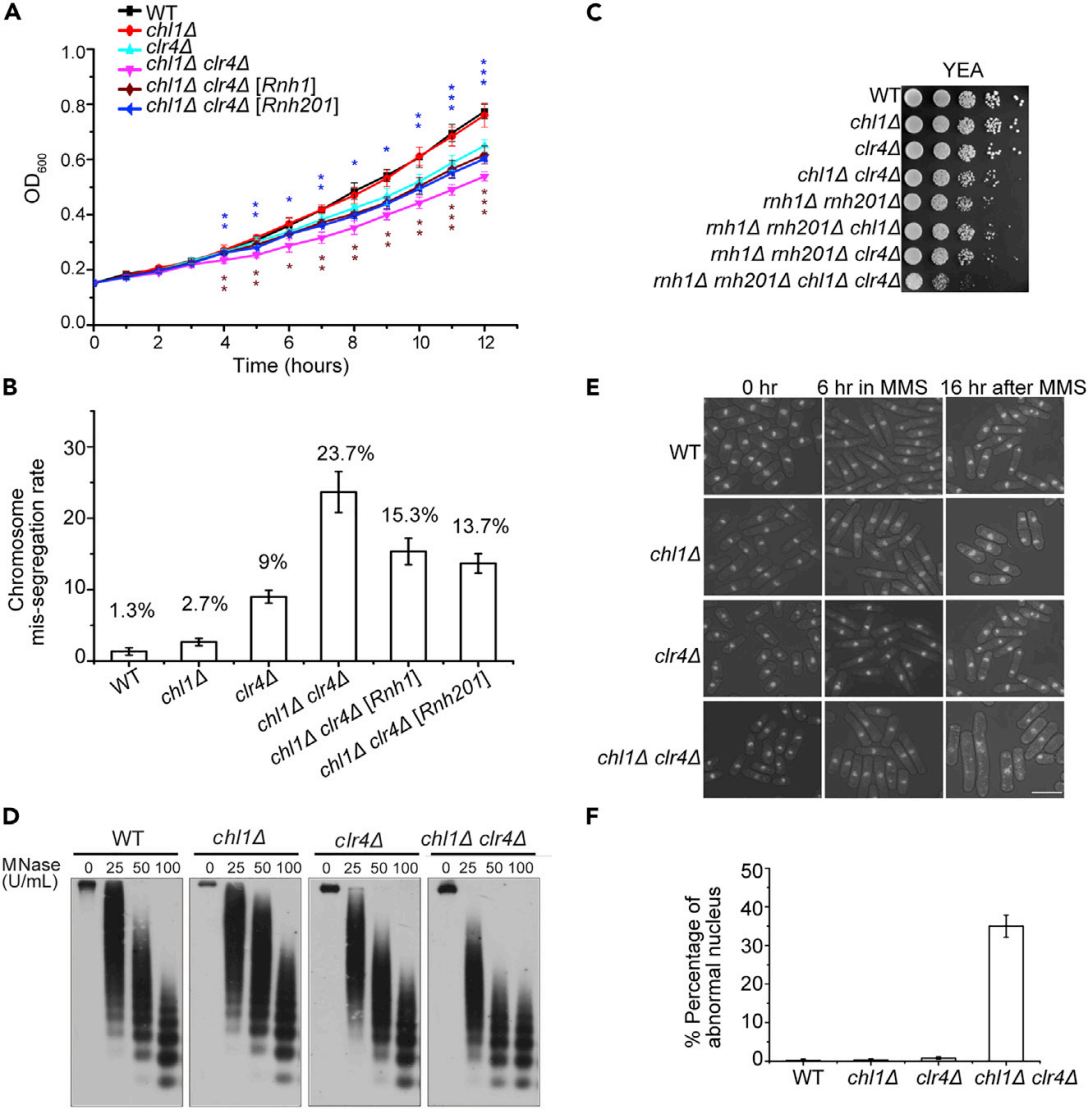
RNA-DNA hybrids produced for *chl1Δclr4Δ* cells impair the genome stability (A) Growth curves of indicated strains transformed with an empty vector and a vector harboring Rnh1 or Rnh201. Values are displayed as mean ± SEM of three biological replicates. The asterisks displayed overexpression of Rnh1 or Rnh201 in *chl1Δclr4Δ* can remarkably recover the growth rate of the strains from 5 to 12 h (∗p < 0.05, ∗∗p < 0.01, ∗∗∗p < 0.001). (B) Percentages of lagging chromosomes in the indicated strains transformed with an empty vector and a vector harboring Rnh1 or Rnh201. (C) Ten-fold serial dilution assays of strains were performed to measure the growth of WT and mutants on YEA. (D) Chromatins from WT and three mutants were digested with the indicated concentrations of micrococcal nuclease (MNase), followed by Southern blot with probe that hybridized to *dh* repeats. (E) Cells were treated with 0.008% MMS for 6 h, washed, and further allowed to grow at 30°C for 16 h in YEA medium without MMS. Samples were fixed with 70% ethanol and stained with DAPI. Scale bar: 10 μm. (F) About 500 cells from (E) for each strain were counted and the proportion of cells with aberrant nuclear morphology was plotted. Error bars represent ±SEM of three biological replicates.

To determine whether accumulation of RNA-DNA hybrid is responsible for the slow growth of *chl1Δ clr4Δ* cells, we compared the growth rates of strains transformed with an empty vector or a vector harboring Rnh1 or Rnh201. The *chl1Δ clr4Δ* mutant overexpressing Rnh1 or Rnh201 had a growth rate comparable to that of *clr4Δ* (Figure 3A), which were significantly faster than *chl1Δ clr4Δ* cells. These data suggest that the difference in growth rates between *clr4Δ* and *chl1Δ clr4Δ* resulted from RNA-DNA hybrid accumulation. In contrast, the minor slow-growth phenotype of *clr4Δ* was not relevant to RNA-DNA hybrids and might be due to failure in association with the cohesin complex. More importantly, overexpression of Rnh1 or Rnh201 dramatically reduced the ratio of lagging chromosomes in *chl1Δ clr4Δ* cells (Figure 3B), suggesting that chromosome segregation defects are caused by RNA-DNA hybrid accumulation. Conversely, a serial dilution plating assay showed that the *rnh1Δ rnh201Δ chl1Δ clr4Δ* quadruple-deletion strain displayed a severe growth defect relative to the other strains (Figure 3C), implying that they had redundancy in the repression of RNA-DNA hybrids. We inferred that endogenous RNase H enzymes are not effective enough for processing all RNA-DNA hybrids produced from the pericentromeric regions in *clr4Δ*. The rest of unprocessed RNA-DNA hybrids can be unwound by Chl1. These results suggest that RNase H and helicase coordinately process RNA-DNA hybrids at specific chromatin regions.

In addition to cohesin attachment, chromatin compaction is also required for proper chromosome segregation. Studies have shown that R-loops associate with DNase I hyperaccessibility, chromatin decompaction, and lower nucleosome occupancy (Powell et al., 2013; Sanz et al., 2016). Because the cotranscriptional RNA-DNA hybrid and the displaced ssDNA can form an R-loop, we investigated whether RNA-DNA accumulation had effects on the chromatin structure at pericentromeric regions. We digested the chromatins from WT, *chl1Δ*, *clr4Δ*, and *chl1Δ clr4Δ* cells with different concentrations of micrococcal nuclease and performed Southern blotting with a probe that hybridized to *dh* repeats. The chromatin of double-deletion strain at the *dh* repeats was more easily digested by the enzyme than that of both WT and two single-deletion strains (Figure 3D). This observation implies that the increased RNA-DNA hybrids interrupt the chromatin compaction, which leads to the defects in chromosome segregation. At same time, we found that *chl1Δ clr4Δ* cells failed to maintain the integrity of nuclei after recovery from genotoxic drug methanesulfonate (MMS) treatment. Four strains were grown in liquid medium containing 0.008% MMS for 6 h, washed, and further inoculated into medium without MMS for 16 h. During this process, cells from each step were fixed and stained with DAPI. After 16 h recovery from MMS treatment, 35% of *chl1Δ clr4Δ* cells displayed diffuse or fragmented nuclei, suggesting that deletion of both Chl1 and Clr4 resulted in failure of recovery from MMS-induced DNA lesions (Figures 3E and 3F).

Chl1 of budding yeast associates with Ctf4 and Ctf7/Eco1 to play a role in sister-chromatid cohesion (Samora et al., 2016; Skibbens, 2004), but here, we did not detect direct interaction of Chl1 with Mcl1 or Eso1, the fission yeast homologs of Ctf4 and Ctf7/Eco1, by a yeast two-hybrid assay (data not shown). Meanwhile, the Chl1 single-deletion mutant did not exhibit severe chromosome segregation defects (Figures 1C and 1D). These data suggest that fission yeast Chl1 does not function in sister chromatid cohesion but is implicated in maintaining a more condensed chromatin at pericentromeric regions by removing RNA-DNA hybrids in the absence of Clr4, which contributes to the genome integrity.

### Ssb1 is responsible for the recruitment of Chl1 to chromatin

**Figure 4:**
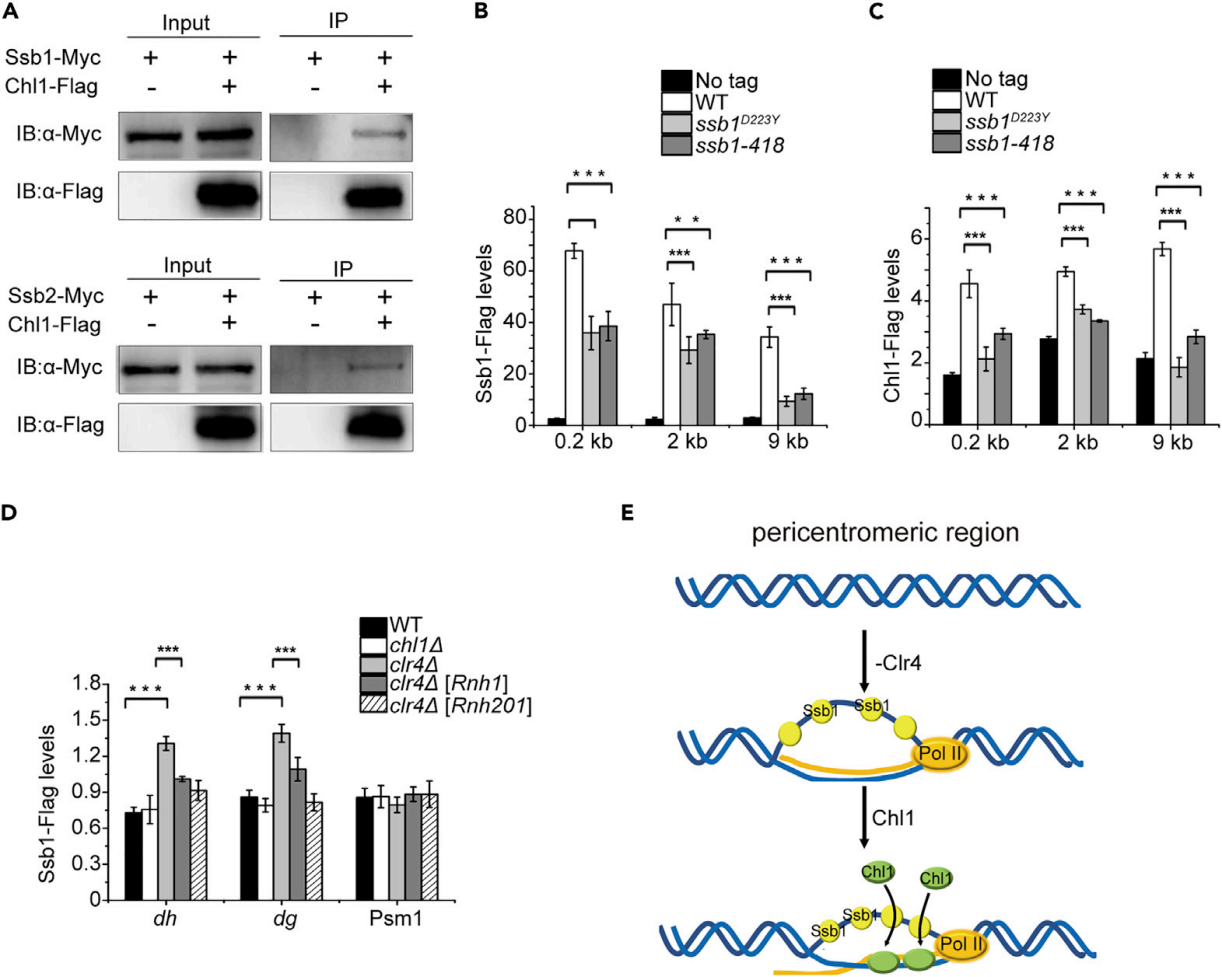
The recruitment of Chl1 depends on Ssb1 (A) Proteins prepared from the indicated strains were immunoprecipitated with anti-Flag resin and analyzed by western blot with an α-myc antibody. (B and C) ChIP-qPCRs were performed to detect the enrichment of Ssb1 (B) or Chl1 (C) at 0.2 kb, 2 kb, and 9 kb from induced DSB in the indicated strains. Error bars represent ±SE of three technical replicates from one ChIP. Three biological replicates were done with the similar results. (D) Ssb1 localization at *dg* and *dh* repeats in the indicated strains transformed with an empty vector and a vector harboring Rnh1 or Rnh201. Error bars represent ±SEM of three technical replicates from one experiment. Three biological replicates were performed. (E) A model in which Chl1 is recruited by Ssb1 that binds single-stranded DNA generated from pericentromeric repeats in the absence of Clr4 to resolve RNA-DNA hybrids.

To elucidate the regulation of Chl1, we performed affinity purification of Chl1-Flag and identified two subunits of RPA by mass spectrometry analysis, namely, Ssb1 and Ssb2 (Figure S2). RPA is a heterotrimeric complex that binds to ssDNA, has versatile roles in DNA repair, replication, and recombination, and exhibits histone assembly ability. The fission yeast RPA consists of three subunits: Ssb1/Rpa1/Rad11, Ssb2/Rpa2, and Ssb3/Rpa3 (Ishiai et al., 1996). To confirm the interactions, we coexpressed Ssb1-Myc or Ssb2-Myc with Chl1-Flag at their native genomic loci and demonstrated that Chl1 coimmunoprecipitated with both Ssb1 and Ssb2 (Figure 4A).

A proteome-wide screen showed that foci formation of Chl1 at induced DNA double-stranded break (DSB) occurred later than that of Ssb1(Yu et al., 2013); therefore, we proposed that Chl1 was recruited by RPA subunit Ssb1 to perform its function. We introduced Ssb1-Flag into a strain containing the HO nuclease recognition site and HO gene integrated into the genome under the control of an inducible nmt1 promoter and then examined the localization of Ssb1 at the HO-induced DSB site in the presence and absence of Chl1. ChIP analysis showed that deletion of Chl1 did not affect Ssb1 accumulation at 0.2 kb, 2 kb, and 9 kb from the break site, suggesting that Ssb1 localization is independent of Chl1 (Figure S3). Deletion of Ssb1 is lethal for fission yeast, but two temperature-sensitive mutants, *ssb1*^*D223Y*^ and *ssb1-418* (Akai et al., 2011; Ono et al., 2003), reduced Ssb1 accumulation at these sites (Figure 4B). Therefore, we checked whether these mutations affected the targeting of Chl1 at DNA break sites. ChIP assays demonstrated that Chl1 levels decreased at these three sites in the two Ssb1 mutants (Figure 4C). This result indicates that Chl1 localization depends on Ssb1. Consistent with the enrichment of Chl1 at *dg* and *dh* repeats in *clr4Δ* cells (Figure 2C), the accumulation of Ssb1 was also observed at these regions in the absence of Clr4, but not in *chl1Δ* cells, whereas overexpression of Rnh1 or Rnh201 repressed the localization of Ssb1 (Figure 4D). Because it has been reported that RPA complex binds to the ssDNA in the R-loops (Nguyen et al., 2017), our data suggest that the ssDNA in the three-stranded structure produced from transcription at *dg* and *dh* repeats is responsible for the recruitment of Ssb1. Collectively, we propose that Chl1 can be recruited to pericentromeric regions by Ssb1 to process RNA-DNA hybrids in *clr4Δ* cells, which protects genome integrity.

## Discussion

In comparison to RNase H enzymes that degrade RNAs in RNA-DNA hybrids, the physiological functions of helicases are less clear. In this study, we found that double deletion of the helicase Chl1 and histone H3K9 methyltransferase Clr4 reduced cell viability (Figure 1B). Chl1 is involved in alleviating the accumulation of RNA-DNA hybrids and maintaining chromatin compaction at pericentromeric regions in *clr4Δ* cells (Figures 2B and 3D), which are required for genome stability.

Centromeric R-loops drive the mitosis-specific ATR pathway to promote proper chromosome segregation (Kabeche et al., 2018). R-loop is composed of an RNA-DNA hybrid and a displaced ssDNA, so elimination of RNA-DNA hybrids contributes to the repression of chromosome missegregation. However, how cells prevent the negative impacts of RNA-DNA hybrids on chromosome segregation is still not very clear. R-loops have been detected at pericentromeric heterochromatin regions in maize (Liu et al., 2021), but immunostaining with an S9.6 antibody showed that the percentage of RNA-DNA hybrids at heterochromatic regions is very low compared with that at euchromatic regions in *S. pombe* (Nakama et al., 2012). Consistent with this result, there was no significant enrichment of RNA-DNA hybrids at *dg* and *dh* repeats in asynchronized WT cells (Figure 2B), which could be due to the fact that heterochromatin represses transcription in fission yeast. The roles of heterochromatin in cohesion and chromosome segregation have been extensively studied (Bernard et al., 2001; Gartenberg, 2009; McKinley and Cheeseman, 2016). Intact heterochromatin ensures proper sister chromatid cohesion and represses transcription at the heterochromatic region. Depletion of H3K9 methylation abolishes the attachment of cohesins, whereas Chl1 can partially suppress chromosome segregation defects by removing RNA-DNA hybrids that affect the chromatin state at *dg* and *dh* repeats and therefore increase cell viability. Moreover, the evidence that overexpression of two RNase H enzymes complements the growth defect of *chl1Δ clr4Δ* cells also confirms that the slow-growth phenotype results from RNA-DNA hybrid accumulation (Figure 3A). Decreased ratios of lagging chromosomes in overexpressed strains indicate that chromosome segregation defect is one of the reasons for the low viability of *chl1Δ clr4Δ* cells (Figure 3B). Thus, the less condensed pericentromeric heterochromatin structure brought about by RNA-DNA hybrids prevent faithful segregation of sister chromatids during mitosis. Considering that chromatin needs to be restored to its original state after DNA repair or replication (Czaja et al., 2012), more accessible chromatin at *dg* and *dh* repeats caused by double deletion of Chl1 and Clr4 may prevent DNA damage recovery, leading to genome instability and cell death (Figures 3E and 3F). In contrast to Chl1 of budding yeast, single deletion of fission yeast Chl1 does not confer obvious defect in chromosome segregation (Figures 1C and 1D), suggesting that its function is distinct from that in budding yeast.

Although our study focused on the function of Chl1 at *dg* and *dh* repeats of pericentromeric regions, evidence also suggested that Chl1 may function in DNA repair due to its recruitment to the induced DSBs by Ssb1(Figure 4C). However, because *chl1Δ* cells are insensitive to DNA damage reagents, we believe that Chl1 does not play a major role in DNA repair. A previous working model based on the colocalization of RPA with R-loops suggests that RPA binds the displaced ssDNA of R-loop and interacts with RNase H1, which can recognize and degrade RNAs in RNA-DNA hybrids (Nguyen et al., 2017). Furthermore, centromeric R-loop is shown to be covered with RPA (Kabeche et al., 2018). Our study demonstrates the recruitment of Ssb1 at highly transcribed pericentromeric regions partially depends on the accumulation of RNA-DNA hybrids (Figure 4D). Hence, we propose here that RPA subunit Ssb1 associates with the displaced ssDNA of RNA-DNA hybrid and facilitates recognition of RNA-DNA hybrids by Chl1, which is able to remove this three-stranded structure by resolving RNA-DNA hybrids and alleviate chromosome segregation defects in the *clr4Δ* mutant (Figure 4E). Certainly, repression of transcription by Clr4 is an alternative way to prevent the generation of RNA-DNA hybrids. At this time, the cohesin complex does not attach to heterochromatin through Swi6 without H3K9 methylation, which results in chromosome missegregation and cannot be rescued by Chl1. These results suggest that Chl1 and Clr4 work together to sustain genome integrity by reducing the formation of RNA-DNA hybrids at pericentromeric regions.

### Limitations of the study

One of the limitations of this study is that the current evidence is insufficient to determine whether accumulation of RNA-DNA hybrids at heterochromatin is the only reason for genome instability. Chl1 localized at DNA double-stranded break after Ssb1, so its role in RNA-DNA hybrids processing might also have a small contribution to genome integrity outside of heterochromatin. Moreover, Chl1 also has helicase activity on DNA-DNA hybrids. Our study did not address the question of whether the DNA helicase activity of Chl1 is involved in the maintenance of chromatin compaction and cell survival. In addition to Clr4, deletions of several components in CLRC complex and factors implicated in RNAi pathway lead to the loss of histone H3K9 methylation and increased transcription at heterochromatin as well. More experiments are required to reveal whether Chl1 can repress the RNA-DNA hybrids that generate from highly transcribed heterochromatin in the mutants of these genes.

## STAR★Methods

### Key resources table

**Table:** 

REAGENT or RESOURCE	SOURCE	IDENTIFIER
**Antibodies**		

Rabbit polyclonal anti-Flag	Sigma Aldrich	Cat# F7425; RRID:AB_439687
Rabbit polyclonal anti-Myc	Cell Signaling Technology	Cat# 2278; RRID:AB_490778
Mouse S9.6 anti RNA-DNA hybrid	Millipore	Cat# MABE1095; RRID:AB_2861387
Rat monoclonal anti-tubulin	Abcam	Cat# ab6160; RRID:AB_305328
goat polyclonal anti-rat	Abcam	Cat# ab150157; RRID:AB_2722511

**Bacterial and virus strains**		

BL21(DE3) *E. coli*	Thermo Fischer	Cat# EC0114

**Chemicals, peptides, and recombinant proteins**		

DAPI	Sigma Aldrich	Cat# D9542
Proteinase K	Thermo Fisher	Cat# EO0491
RNase A	Thermo Fisher	Cat# EN0531
Micrococcal nuclease	Roche	Cat# 10107921001
proteinA+G magnetic beads	Millipore	Cat# 16-663
Anti-Flag M2 affinity gel	Sigma Aldrich	Cat# A2220
Protease Inhibitor Cocktail	Roche	Cat# RE04693132001
*Hind*III	NEB	Cat# R0104
*Ssp*I	NEB	Cat# R0132
*Xba*I	NEB	Cat# R0145
*Bsr*GI	NEB	Cat# 0575
*EcoR*I	NEB	Cat# R0101
HU (Hydroxyurea)	Sigma Aldrich	Cat# H8627
Lyticase	Sigma Aldrich	Cat# L2524
KOD-plus-Neo	TOYOBO	Cat# KOD-401
G418 Sulfate	VWR	Cat# E859
Paraformaldehyde	Sigma Aldrich	Cat# P6148
Protease Inhibitor Cocktail	Roche	Cat# RE04693132001
SYBR Green Master Mix	Takara	Cat# RR420A

**Critical commercial assays**		

Maxima H Minus cDNA Synthesis	Thermo Fisher	Cat# M1682
Master Mix with dsDNase RNeasy Mini Kit	Qiagen	Cat# 74104
Random Primer DNA Labeling Kit	Takara	Cat# 6045

**Deposited data**		

Mass spectrometry proteomics data	This study	

**Experimental models: Organisms/strains**		

Strains are summarized in Table S1	This paper	N/A

**Oligonucleotides**		

Real-time PCR primers are summarized in Table S2	This paper	N/A

**Software and algorithms**		

Adobe Illustrator CS6	Adobe System Software	https://www.adobe.com/cn/products/illustrator.html
Origin8.0	Origin System Software	https://www.origin.com/hkg/enus/store/download
IBM SPSS Statistics 20	IBM SPSS Statistics Software	https://www.ibm.com/cn-zh/products/spss-statistics
Adobe Photoshop CC	Adobe Photoshop System Software	Official Adobe Photoshop | Photo and design software

### Resource availability

#### Lead contact

Further information and requests for resources should be directed to and will be fulfilled by the lead contact, Yu Wang (yw2250@cau.edu.cn).

#### Materials availability

All newly created strains generated in this study are available upon request.

### Experimental model and subject details

#### Yeast strains and cell cultures

Yeast strains harboring deleted or epitope-tagged genes were constructed using a PCR-based module method (Bahler et al., 1998; Wach et al., 1994). Genetic crosses were used to construct double mutants. Cell used in this study were cultured in YEA at 30°C unless otherwise noted.

### Method details

#### HO induction

HO induction was performed as described previously. Cells were first cultured in EMM-Leu supplemented with thiamine at 30°C and then shifted to thiamine-free EMM-Leu for 22 h to induce DSB. For HO induction of temperature-sensitive strains, Cells were shifted to 37°C for 3.5 h after incubated at 30°C for 22 h, and then used for ChIP assay.

#### Growth curves

The overnight cultures were diluted to an OD_600_ of ∼ 0.1 as the initial value of the growth curve. Cells were further grown for 12 h with agitation and OD_600_ was measured hourly.

#### Dilution analysis

Logarithmic phase cells were normalized to an OD_600_ of 0.5. Ten-fold serial dilutions were spotted onto YEA plates and YEA with MMS (Sigma Aldrich). Cells were grown at 30°C for 3-4 days.

#### HU synchronization assay

Exponentially growing cells were synchronized in S phase by being treated with 15 mM HU (Sigma) for 4.5 h. Samples were released by washing twice in hydroxyurea-free media and then re-inoculated into medium lacking HU for further grown. Ten OD units of cells were collected every 30 min after release from HU block. At the same time, 1 ml of sample was collected, fixed with 70% ethanol and stained with calcofluor. Cell cycle progression was monitored by microscopically counting septated cells.

#### Microscopy

Cells were grown to mid-log phase and then treated with 0.008% MMS (Sigma) for 6 h. Samples were collected and washed twice with medium lacking MMS and then re-inoculated into medium lacking MMS. Cells were collected at 16 h after removing MMS and fixed with 70% ethanol. DAPI stained cells were photographed using an Olympus BX53 microscope. Cells with aberrant nuclear morphology were counted and graph was plotted.

For visualization of tubulin by immunofluorescence microscopy, the mid-log phase cells were treated at 18°C for 5 min, and then fixed with 3% paraformaldehyde (Sigma Aldrich) at 18°C for 30 min. Cells were washed twice with PEM buffer (100 mM PIPES, pH 6.9, 1 mM EGTA, 1 mM MgSO_4_) and twice with PEMS buffer (100 mM PIPES, pH 6.9, 1 mM EGTA, 1 mM MgSO_4_, 1.2 M Sorbitol), followed by digestion for 30 min at 37°C with 0.5 mg/ml lyticase in PEMS buffer. Cells were washed twice with PEM buffer, and then incubated at 4°C overnight h in PEMBAL buffer (100 mM PIPES, pH 6.9, 1 mM EGTA, 1 mM MgSO_4_, 1% BSA, 100 mM Lysine, 0.1% NaN_3_) with anti-tubulin monoclonal immunoglobulin G (IgG) (Abcam). Cells were washed 3 times with PEMBAL buffer, and then resuspended in PEMBAL buffer containing Alexa Fluor 488 goat anti-rat IgG (Abcam). Samples were washed 3 times with PEMBAL buffer and DAPI-stained DNA was used to visualize nuclei. Images were photographed using an Olympus BX53 microscope.

For methylene blue staining assay, exponentially growing cells were suspended in 0.01% methylene blue solution for 10 minutes, and then cell color was observed using an Olympus BX53 microscope.

#### Chromosome loss assay

Cells with minichromosome Ch16 were grown to log phase, samples were diluted and then plated on medium lacking adenine. The number of colonies with half red and half white were counted and chromosome loss was calculated according to the following formula: loss rate =[n / (n+N)]×100%, where n is the number of colonies with half red and half white color and N is the number of colonies with white color.

#### Western blot

Cells were resuspended in chip lysis buffer (50 mM HEPES, pH 7.5, 140 mM NaCl, 1% Triton X-100, 0.1% Deoxycholate, Roche protease inhibitor, 1 mM PMSF) and lysed with glass beads. After centrifugation, the supernatant was collected. 2×SDS buffer was added into an equal volume of samples and incubated for 5 min at 100°C. A rabbit polyclonal anti-Myc antibody (CST) and a rabbit polyclonal anti-Flag antibody (Sigma) antibody were used for Western blot analyses.

#### Co-immunoprecipitation (Co-IP)

Protein purification were performed as described previously with some modifications (Wang et al., 2009). Ten liters of overnight cultured cells were harvested, washed with PBS buffer (100 mM NaCl, 10 mM Na_2_HPO_4_⋅12H_2_O, 2 mM KH_2_PO_4_) and frozen in liquid nitrogen. Cells were lysed with a grind instrument (Jinxin, Tiss-24) and then resuspended in HC buffer (150 mM HEPES-KOH, pH 7.6, 1 mM EDTA, 250 mM KCl, 10% glycerol, 1 mM DTT, Roche protease inhibitor, 1 mM PMSF). The supernatants were collected after centrifugation at 12, 000×g for 3 h, followed by incubation with anti-Flag M2 affinity agarose beads (Sigma) for 3 h. Beads were washed six times with HC buffer and two times with PBS buffer. The protein complex was eluted with 100 mg/ml 3×Flag peptide and sent for mass spectrometry analysis. A small number of beads were boiled in SDS buffer for Western blot.

#### ChIP

Exponentially growing cells were fixed with 3% formaldehyde for 30 min at 18°C. Cells were harvested, resuspended in chip lysis buffer and lysed with glass beads. Cell lysate was sonicated to obtain DNA fragment between 600-1000 bp. The supernatants were collected after centrifugation at 10, 000×g for 15 min at 4°C. Anti-Flag M2 affinity agarose beads were used for immunoprecipitation. After incubation at 4°C for overnight, chromatin bound agarose beads were harvested and washed twice with chip lysis buffer, once with chip lysis buffer containing 0.5 M NaCl, once with wash buffer (10 mM Tris-HCl, pH 8.0, 0.25 M LiCl, 0.5% NP-40, 0.5% sodium deoxycholate, 1 mM EDTA) and once with TE buffer. After crosslinking was reversed in TES (50 mM Tris-HCl, pH 8.0, 10 mM EDTA, 1% SDS) at 65°C for overnight, immunoprecipitated chromatin and input DNA were digested with 8 μg of RNase A for 1 h at 37°C and 10 μg of proteinase K for 5 h at 55°C, followed by phenol/chloroform/isoamylol (25:24:1) extraction. DNA was precipitated by ethanol with 35 μl of 3 M sodium acetate and resuspended in TE buffer. Purified DNA was used for RT-PCR analysis with SYBR Green Master Mix (Takara).

#### DRIP

ChIP analysis was performed as described previously with some modifications (Taneja et al., 2017). The cells were lysed by 0.5 mg/ml lyticase for 45 min at 37°C in PEMS. The spheroplasts were collected and resuspended with TE buffer containing 1% SDS, and then incubated for 10 min at 65°C. The chromatin was obtained by adding 5 M potassium acetate, followed by centrifugation at 10000 x g for 15 min at 4°C. Chromatin fractions dissolved in TE buffer were precipitated with equal volume of isopropanol. The pellets were digested with 5 μg of RNase A for 1 h at 37°C, followed by 10 μg of proteinase K for 5 h at 55°C. DNA was purified by phenol/chloroform/isoamylol (25:24:1) extraction, and then precipitated by ethanol with 35 μl of 3 M sodium acetate. The pellet was dissolved in TE buffer and digested with 50 U of *EcoRI*, *HindIII*, *BsrGI*, *SspI* and *XbaI* with 2.5 mg of BSA. Half of each sample was digested with 8 μl of RNase H (NEB) at 37°C overnight. DNA was extracted with phenol/chloroform/isoamylol (25:24:1), precipitated by ethanol with 35 μl of 3 M sodium acetate and dissolved in binding buffer (140 mM NaCl, 10 mM Na_2_HPO_4_, 0.05% Triton X-100). The DNA:RNA was immunoprecipitated with S9.6 antibody in binding buffer at 4°C for 16 h. Protein A+G magnetic beads (Millipore) were used for recovering immunoprecipitated DNA. DNA was washed four times with binding buffer, and then eluted with elution buffer (10 mM EDTA, pH 8.0, 50 mM Tris-HCl pH 8.0, 0.5% SDS). Immunoprecipitated DNA was treated with Proteinase K and purified by phenol/chloroform/isoamyl extraction. Purified DNA was dissolved in TE buffer and used for RT-PCR.

#### Helicase assays

Helicase reactions (20 μl) were performed by incubating 100 ng, 50 ng or 25 ng of Recombinant GST-Chl1 purified from *E. coli* BL21 cells with 0.05 nM duplex RNA/DNA substrate at 30°C for 10 min in reaction buffer containing 25 mM Tris-HCl pH 7.5, 2 mM MgCl_2_, 2 mM DTT, 2 mM ATP and 0.25 mg/mL BSA. The sequence of RNA of RNA/DNA duplex is 5^’^-CAUUUUGCUGCCGGUCACG-3’ and the sequence of DNA is 5’-CGTGACCGGCAGCAAAATGACCCTTTTTTTTTTTTTTTTTTTTTTTTTTTTTTTTTTT-3’. The RNA was biotin-labeled. The reactions were terminated by adding 4 μl of stop solution (60 mM EDTA, pH 8.0, 40% sucrose, 0.25% bromophenol blue and 0.25% xylene cyanol). The products were separated by electrophoresis on a 12% nondenaturing polyacrylamide gel in 0.5×TBE buffer, transferred to nylon membrane and crosslinked 2 min by UV. The biotin-labeled signals were detected by the Chemiluminescent Nucleic Acid Detection Module kit.

#### Micrococcal nuclease digestion of chromatin

Ten OD units of cells were harvested and washed with SP1 buffer (20 mM citrate/phosphate, 40 mM EDTA). The cells were digested by Zymolyase-100T in SP2 buffer (50 mM citrate/phosphate, 1.2 M sorbitol) and washed with SP3 buffer (10 mM Tris-HCl, 1.2 M sorbitol). The pellet was resuspended in NDB buffer (10 mM Tris-HCl, 1.2 M sorbitol, 5 mM MgCl_2_, 1 mM CaCl_2_) and divided into four portions. Three of them were digested with 25 U/ml、50 U/ml、100 U/ml of MNase for 5 min, respectively. The reactions were stopped by adding 50 mM EDTA and then treated with Proteinase K and RNase A. DNA was purified with isometric phenol/ chloroform/isoamylol extraction and precipitated by ethanol with 35 μl of 3 M sodium acetate. The purified DNA was dissolved in TE buffer, separated by agarose gel electrophoresis with 1% (w/v) agarose in TAE buffer for 16 h at 40 volts and transferred to Nylon membranes. Afterward, the membrane was washed with 2×SSC buffer (300 mM NaCl, 30 mM sodium citrate, pH 7.0). DNA probe was labeled with ^32^P using random primer DNA Labeling Kit (Takara) and denatured by boiling for 5 min, followed by cooling on ice for 5 min. The membrane was incubated with probe for overnight at 65°C in hybridization buffer (7% SDS, 1 mM EDTA, 0.25 M Na_2_HPO_4_, pH 7.4) with rotation. The membrane was washed three times with wash buffer (1% SDS, 1 mM EDTA, 0.1 M Na_2_HPO_4_, pH 7.4). Image was obtained using autoradiography.

### Quantification and statistical analysis

Statistical analyses in this paper were performed with Origin 8.5 software for Windows. All data were calculated by Student’s t-test and presented as mean±SEM (standard deviation of the mean). p values less than 0.001, 0.01, 0.05 and were assigned with ∗∗∗, ∗∗ and ∗, respectively.

## Data Availability

All data reported in this paper will be shared by the lead contact upon request. This paper does not report original code. Any additional information required to reanalyze the data reported in this paper is available from the lead contact upon request.
