# Supplementary material for: Chl1 coordinates with H3K9 methyltransferase Clr4 to reduce the accumulation of RNA-DNA hybrids and maintain genome stability
Source: iScience. 2022 Apr 27;25(5):104313. doi: 10.1016/j.isci.2022.104313 (PMC9118164; doi:10.1016/j.isci.2022.104313)
Supplement: Document S1. Figures S1–S3 and Tables S1 and S2 [file mmc1.pdf]

iScience, Volume *25*

Supplemental information

Chl1 coordinates with H3K9 methyltransferase Clr4 to reduce the accumulation of RNA-DNA hybrids and maintain genome stability

Deyun He, Yazhen Guo, Jinkui Cheng, and Yu Wang

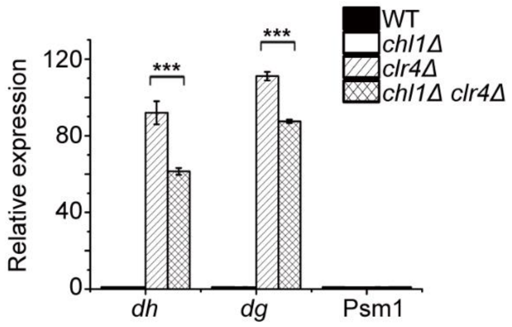

Figure S1. Transcripts of *dg*, *dh* and Psm1 in the indicated strains, related to Figure 2.

Protein	MV	Spectra count	Percent coverage
Ssb1	68610	11	28%
Ssb2	30372	3	20%

Figure S2. Mass spectrometry analysis of proteins associated with Chl1-Flag, related to Figure 4.

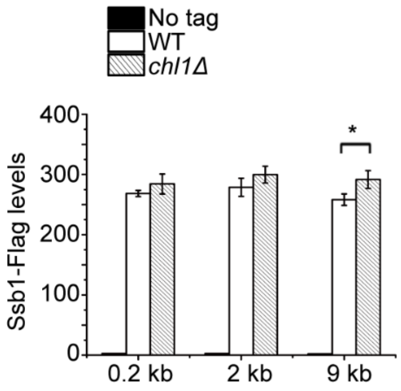

Figure S3. Localization of Ssb1 at HO-induced DSBs in the indicated strains, related to Figure 4.

Table S1. Yeast strains used in this study, related to STAR Methods.

Strains	Genotype	Source
LLD3650,	*h- his3D1 ura4-D18 crb2Δ::ura4 Rad22-CFP::KanMX6 arg3::HOsite-KanMX4 leu1-32::YFP-Crb2::leu1 nmt41-HO-his3*	(Du et al., 2006)
ywp746,	*h- his3D1 ura4-D18 crb2Δ::ura4 Rad22-CFP::KanMX6 arg3::HOsite-KanMX4 leu1-32::YFP-Crb2::leu1 nmt41-HO-his3 Chl1-flag::HphMX6*	This study
SPJ1577,	*h+ Leu1-32 ura4DS/E*	(Wang et al., 2009)
ywp168,	*h+ Leu1-32 ura4DS/E chl1Δ::HphMX6*	This study
ywp702,	*h+ leu1-32 ura4 DS/E Chl1-5xflag::KanMX6*	This study
ywp1108,	*h+ leu1-32 ura4 DS/E Ssb1-13xmyc::NatMX6 Chl1-5xflag::HphMX6*	This study
ywp1112,	*h+ leu1-32 ura4 DS/E Ssb2-13xmyc::KanMX6 Chl1-5xflag::HphMX6*	This study
ywp1106,	*h+ leu1-32 ura4 DS/E Ssb1-13xmyc::NatMX6*	This study
ywp1111,	*h+ leu1-32 ura4 DS/E Ssb2-13xmyc::KanMX6*	This study
ywp952,	*h- his3D1 ura4-D18 crb2Δ::ura4 Rad22-CFP::KanMX6 nmt41-HO-his3 arg3::HOsite-KanMX4 leu1-32::YFP-Crb2::leu1 Ssb1-5xflag::HphMX6*	This study
ywp1005,	*h- his3D1 ura4-D18 crb2Δ::ura4 Rad22CFP::KanMX6 nmt41-HO-his3 arg3::HOsite-KanMX4 leu1-32::YFP-Crb2::leu1 Ssb1-5xflag::HphMX6 chl11Δ::NatMX6*	This study
ywp1328,	*h- his3D1 ura4-D18 crb2Δ::ura4 Rad22-CFP::KanMX6 nmt41-HO-his3 arg3::HOsite-KanMX4 leu1-32::YFP-Crb2::leu1* $ssb1^{D223Y}$*-5xflag::HphMX6*	This study
ywp1330,	*h- his3D1 ura4-D18 crb2Δ::ura4 Rad22-CFP::KanMX6 nmt41-HO-his3 arg3::HOsite-KanMX4 leu1-32::YFP-Crb2::leu1 ssb1-418-5xflag::HphMX6*	This study
ywp1334,	*h- his3D1 ura4-D18 crb2Δ::ura4 Rad22-CFP::KanMX6 nmt41-HO-his3 arg3::HOsite-KanMX4 leu1-32::YFP-woCrb2::leu1* $ssb1^{D223Y}$*::HphMX6 Chl1-5xflag::NatMX6*	This study
ywp1336,	*h- his3D1 ura4-D18 crb2Δ::ura4 Rad22-CFP::KanMX6 nmt41-HO-his3 arg3::HOsite-KanMX4 leu1-32::YFP-Crb2::leu1 Ssb1-418::HphMX6 Chl1-5xflag::NatMX6*	This study
ywp236,	*Mat1Msmto leu1-32 his2 ura4 DS/E clr4Δ::NatMX6*	This study
ywp238,	*Mat1Msmto leu1-32 his2 ura4 DS/E clr4Δ::NatMX6; chl1Δ:: KanMX6*	This study
ywp863,	*h+ Leu1-32 ura4DS/E Ssb1-5xflag::KanMX6*	This study
ywp1173,	*h+ Leu1-32 ura4DS/E Ssb1-5xflag::KanMX6 chl1Δ::KanMX6*	This study
ywp1175,	*h+ Leu1-32 ura4DS/E Ssb1-5xflag::KanMX6 clr4Δ::NatMX6*	This study

Continued

Strains	Genotype	Source
ywp758,	*Mat1Msmto leu1-32 his2 ura4 DS/E Chl1-5xflag::KanMX6 clr4Δ::HphMX6*	This study
ywp1543,	*Mat1Msmto leu1-32 ura4 DS/E rnh1Δ::NatMX6 rnh201Δ::KanMX6*	This study
ywp1545,	*h+ leu1-32 ura4 DS/E rnh1Δ::NatMX6 rnh201Δ::KanMX6 chl1Δ::HphMX6*	This study
ywp1547,	*h+ leu1-32 ura4 DS/E rnh1Δ::NatMX6 rnh201Δ::KanMX6 clr4Δ::HphMX6*	This study
ywp1549,	*h+ leu1-32 ura4 DS/E rnh1Δ::NatMX6 rnh201Δ::KanMX6 chl1Δ::HphMX6 clr4Δ::HphMX6*	This study
ywp1483,	*Mat1Msmto leu1-32 his2 ura4 DS/E pREP41MHN*	This study
ywp1485,	*Mat1Msmto leu1-32 ura4 DS/E chl1Δ::KanMX6 pREP41MHN*	This study
ywp1487,	*Mat1Msmto leu1-32 ura4 DS/E clr4Δ::HphMX6 pREP41MHN*	This study
ywp1489,	*Mat1Msmto leu1-32 ura4 DS/E chl1Δ::KanMX6 clr4Δ::HphMX6 pREP41MHN*	This study
ywp1507,	*h+ leu1-32 ura4 DS/E pREP41MHN-Rnh201*	This study
ywp1509,	*h+ leu1-32 ura4 DS/E chl1Δ::HphMX6 pREP41MHN-Rnh201*	This study
ywp1511,	*Mat1Msmto leu1-32 ura4 DS/E clr4Δ::NatMX6 pREP41MHN-Rnh201*	This study
ywp1513,	*Mat1Msmto leu1-32 ura4 DS/E clr4Δ::NatMX6 chl1Δ::KanMX6 pREP41MHN-Rnh201*	This study
ywp1495,	*h+ leu1-32 ura4 DS/E pREP41MHN-Rnh1*	This study
ywp1497,	*h+ leu1-32 ura4 DS/E chl1Δ::HphMX6 pREP41MHN-Rnh1*	This study
ywp1499,	*Mat1Msmto leu1-32 ura4 DS/E clr4Δ::NatMX6 pREP41MHN-Rnh1*	This study
ywp1501,	*Mat1Msmto leu1-32 ura4 DS/E clr4Δ::NatMX6 chl1Δ::KanMX6 pREP41MHN-Rnh1*	This study
SPJ529,	*Mat1Msmto leu1-32 his2 ura4 DS/E Ch16(m23:::ura4 Tel72; ade6-216)*	This study
ywp1441,	*Mat1Msmto leu1-32 ura4 DS/E chl1Δ::KanMX6 Ch16 (m23:::ura4 Tel72; ade6-216)*	This study
ywp1443,	*Mat1Msmto leu1-32 ura4 DS/E clr4Δ::HphMX6 Ch16 (m23:::ura4 Tel72; ade6-216)*	This study
ywp1447,	*Mat1Msmto leu1-32 ura4 DS/E chl1Δ::KanMX6 clr4Δ::HphMX6 Ch16 (m23:::ura4 Tel72; ade6-216)*	This study

Table S2. Real-time PCR primers used in this study Yeast strains used in this study, related to STAR Methods.

Primer	Sequence (5'-3')
Psm1-R	TCCGAGATATGCGAAAAGGC
Psm1-F	CGCAATGACGGTTCTGAGAG
HO-9 kb-R	CTAACAATCAATGGTCATCATCTGGGCCGC
HO-9 kb-F	CAAATACCTCTCGAGCCTTTACTGTTGGCG
HO-2 kb-R	CCAGCAGAAATGTTGAACCCGAGTATAGCG
HO-2 kb-F	GAAATACACCACCTGTAATGGGACGAGCAC
HO-0.2 kb-R	TTCGTACCCAATTCGCCCTATAGTGAGTCG
HO-0.2 kb-F	GCATACGATATATTACGGCGCCAATCTCGC
p33 qPCR-R	CTGTTCGTGAATGCTGAGAAAG
p33 qPCR-F	TATCCTGCGTCTCGGTATCC
p30 qPCR-R	CATCAAGCGAGTCGAGATGA
p30 qPCR-F	CCATATCAATTTCCCATGTTCC
HO-flanking fw	CGACTCACTATAGGGCGAATTGGGTACGAA
HO-flanking rv	CTAAATGTACGGGCGACAGTCACATCATGC
